# Culturable Facultative Methylotrophic Bacteria from the Cactus *Neobuxbaumia macrocephala* Possess the Locus *xoxF* and Consume Methanol in the Presence of Ce^3+^ and Ca^2+^

**DOI:** 10.1264/jsme2.ME17070

**Published:** 2017-09-27

**Authors:** María del Rocío Bustillos-Cristales, Ivan Corona-Gutierrez, Miguel Castañeda-Lucio, Carolina Águila-Zempoaltécatl, Eduardo Seynos-García, Ismael Hernández-Lucas, Jesús Muñoz-Rojas, Liliana Medina-Aparicio, Luis Ernesto Fuentes-Ramírez

**Affiliations:** 1 Instituto de Ciencias, Benemérita Universidad Autónoma de Puebla Edif. 103-J, Ciudad Universitaria, CP 72570, Puebla, Pue. Mexico; 2 Instituto de Biotecnología, Universidad Nacional Autónoma de México Av. Chamilpa 2001, CP 62210, Cuernavaca, Mor. Mexico

**Keywords:** rare-earth elements, lanthanides, pectin metabolism, Tehuacan, *xoxF5*

## Abstract

Methanol-consuming culturable bacteria were isolated from the plant surface, rhizosphere, and inside the stem of *Neobuxbaumia macrocephala*. All 38 isolates were facultative methylotrophic microorganisms. Their classification included the Classes *Actinobacteria*, *Sphingobacteriia*, *Alpha*-, *Beta*-, and *Gammaproteobacteria*. The deduced amino acid sequences of methanol dehydrogenase obtained by PCR belonging to *Actinobacteria*, *Alpha*-, *Beta*-, and *Gammaproteobacteria* showed high similarity to rare-earth element (REE)-dependent XoxF methanol dehydrogenases, particularly the group XoxF5. The sequences included Asp^301^, the REE-coordinating amino acid, present in all known XoxF dehydrogenases and absent in MxaF methanol dehydrogenases. The quantity of the isolates showed positive hybridization with a *xoxF* probe, but not with a *mxaF* probe. Isolates of all taxonomic groups showed methylotrophic growth in the presence of Ce^3+^ or Ca^2+^. The presence of *xoxF*-like sequences in methylotrophic bacteria from *N. macrocephala* and its potential relationship with their adaptability to xerophytic plants are discussed.

Methanol, one of the most common C1 compounds delivered by plants, is released through the stomata. This compound is also produced with the decay of pectin and lignin from dead plant tissue (1, 19, 47). Methanol and organic molecules without C-C bonds are utilized as carbon and energy sources by methylotrophic organisms. These organisms are classified as facultative or obligate methylotrophs depending on their capability to use compounds with multiple C and C-C bonds. Methylotrophic microorganisms are ubiquitous and include organisms of the Classes *Actinobacteria*, *Spirochaetes*, *Alpha*-, *Beta*-, *Gamma*-, and *Deltaproteobacteria*, of the Phyla *Firmicutes*, *Bacteroidetes*, *Chloroflexi*, *Acidobacteria*, *Nitrospirae*, *Verrucomicrobia*, *Cyanobacteria*, and *Planctomycetes*, and even of the domain *Archaea* (5, 8, 15, 22, 25, 29, 30, 35, 38, 43).

Many methylotrophic bacteria are commonly associated with plants. Nevertheless, there have not yet been reports in *Cactaceae*. Several methylotrophs exert positive effects when inoculated in plants (37–39, 54). These responses have been attributed to different mechanisms such as nitrogen fixation, decreased metal toxicity, the contribution of pyrrolo-quinoline quinone (PQQ), elicitation of plant defenses, decreased plant levels of ethylene, and the synthesis of molecules including phytohormones, vitamin B12, polysaccharides, and osmoprotectants (11, 39–41, 49, 57, 60). Methanol and methane-catabolizing microorganisms oxidize methanol through different dehydrogenases, and the methanol dehydrogenase, MxaFI-MDH has been examined in the most detail. It is a heterotetramer that is encoded by the genes *mxaF* and *mxaI*, and its activity depends on PQQ and Ca^2+^ as co-factors (10). MxaFI-MDH is typically carried by *Alphaproteobacteria*, *Gammaproteobacteria*, and a few *Betaproteobacteria*. Some *Betaproteobacteria* also possess the PQQ methanol dehydrogenase MDH2, which shows sequence similarity to MxaFI-MDH (24, Fig. 1). Low GC Gram-positive methylotrophs typically have a NADPH-dependent methanol dehydrogenase (6), and a methanol:NDMA (N,N′-dimethyl-4-nitrosoaniline) oxidoreductase has been reported in the Class *Actinobacteria* (23, 48). Other dehydrogenases phylogenetically related to MxaFI-MDH include a diverse but related group of enzymes called XoxF. Recent studies demonstrated that XoxF dehydrogenases oxidize methanol and depend on rare-earth elements instead of Ca^2+^ as co-factors (18, 27, 46, 50). A sequence analysis revealed that XoxF enzymes are grouped in at least five classes (55).

*Neobuxbaumia macrocephala* is a xerophytic branching columnar *Cactaceae* with a height from 3 to 15 m. This plant is endemic to the Tehuacán-Cuicatlán Biosphere Reserve and its distribution is confined to a few patches with calcareous soils (44, 51, 58). *N. macrocephala* has smaller populations than other *Neobuxbaumia* species that reside in other semi-arid habitats (16).

Rhizospheric and non-rhizospheric bacteria associated with cacti mostly include *Actinobacteria*, *Firmicutes*, *Alphaproteobacteria*, *Cyanobacteria*, *Planctomycetes*, *Bacteroidetes*, *Chloroflexi*, and *Acidobacteria* (2, 3, 34, 56). Limited information is currently available on the ecological interactions among cacti and microorganisms, including those of *N. macrocephala*. In order to design any future restoration strategy for endangered plant species, it is desirable to retrieve a broad knowledge of its biology. The diversity of cultured methylotrophic bacteria associated with this plant was investigated as the first step with the aim of gaining insights into the ecology of *N. macrocephala* with microorganisms, and as a prerequisite for future inoculation experiments using this plant.

## Materials and Methods

### Sampling

Rhizospheric soil, surface, and endophytic samples were obtained from six plant specimens from the Tehuacán-Cuicatlán Biosphere Reserve. Approximately 10 g of rhizospheric soil (profundity 15–25 cm) was retrieved from a distance within 1 m of the sampled specimen. Approximately 5 cm^2^ of the stem surface was sampled with sterile swabs soaked in sterile 10 mM MgSO_4_ solution. The swabs were deposited in 1 mL of the same solution. Regarding endophytic samples, *ca.* 5 cm^2^ of the stem surface was disinfected with 70% ethanol, and *ca.* 1 cm^3^ of tissue was extracted with a sterile scalpel. All samples were kept in sterile plastic sealed bags and transported under chilled conditions to the lab.

### Isolation and DNA extraction

In order to isolate endophytes, approximately 2 mm of surface plant tissues including the cuticle were discarded under sterile conditions. The remaining plant material was macerated in a sterile mortar and resuspended in 10 mM MgSO_4_ (1:10 w:v). Epiphytic suspensions and soil dilutions in 10 mM MgSO_4_ were inoculated on plates (1.6% agar) of methanol mineral salts medium (MMSM; 21) containing 0.5% methanol; 6.89 mM K_2_HPO_4_; 4.56 mM KH_2_PO_4_; 0.228 mM CaCl_2_; 0.811 mM MgSO_4_; 1.71 mM NaCl; 3.7 μM FeCl_3_; 3.8 mM (NH_4_)_2_SO_4_; 20 nM CuSO_4_; 41.5 nM MnSO_4_; 38 nM Na_2_MoO_4_; 0.163 μM H_3_BO_3_; 0.243 μM ZnSO_4_; and 21 nM CoCl_2_, and incubated at 30°C for 8–10 d. Isolated bacterial colonies were streaked in the same medium and incubated at 30°C until growth was observed. Isolated colonies were grown in the same medium and also in GP containing (L^−1^): Casein peptone 10 g, glycerol 10 g, and agar 15 g. DNA was extracted from cells growing in MMSM medium with the DNA Isolation Kit for Cells and Tissues (Roche Diagnostics, Indianapolis, IN, USA) following the recommended instructions of the supplier.

### Ca^2+^ and Ce^3+^-methanol dependent growth

Isolates were grown in GP plates at 30°C for 4 d. One loopful of bacterial cells was washed twice in 10 mM MgSO_4_, resuspended in 10 mL of the same solution, and 5 μL of the suspension was inoculated in 5 mL of modified MMSM with 30 μM CaCl_2_ or lacking Ca^2+^ but with 30 μM CeCl_3_. Cells were incubated at 30°C under shaking for 5 d. Bacterial growth was assessed by absorbance at 600 nm 72, 96, 120, and 144 h after the inoculation. The cultures of three independent replicates grown in either Ca^2+^ or Ce^3+^-MMSM broths were statistically compared by the unpaired *t*-test, *P*<0.05.

### Dot blot hybridization

Genomic DNAs were transferred to nylon filters by dot blots, with 1 μg of DNA per dot, except for *M. extorquens* JCM2802, which had 100 ng. One microgram of *U. maydis* 207 was used as a negative control. One hundred nanograms of DNA ^32^P-labeled probes specific for *mxaF* and *xoxF5* were used for hybridizations. These probes were obtained by the PCR amplification of *Methylobacterium extorquens* JCM2802 genomic DNA with the primers mxa f1003 and mxa r1561 (42); and xoxFf361 and xoxFr603 (Table 1), for *mxaF* and *xoxF5*, respectively. The sizes of the probes were *ca.* 560 bases for *mxaF* and *ca.* 240 bases for *xoxF5*. The probes were labeled with [α-^32^P]dCTP by polymerase extension using random primers (Amersham Rediprime II DNA Labeling System, GE Healthcare, Pittsburgh, PA, USA). Prehybridization and hybridization were performed at 65°C for 12 h using Rapid Hyb buffer (GE Healthcare). The membranes were washed under high stringency conditions (2×SSC [1×SSC is 0.15 M NaCl plus 0.015 M sodium citrate] plus 0.1% SDS for 10 min, 1×SSC plus 0.1% SDS for 15 min, 0.5×SSC plus 0.1% SDS for 15 min, 0.1×SSC plus 0.1% SDS for 15 min, 0.1×SSC plus 0.1% SDS at 65°C for 30 min, and SDS was then removed with 0.1×SSC) (52).

### DNA amplification and sequencing

16S rRNA genes were amplified with the primers B27F (5′-TAG AGT TTG ATC CTG GCT CAG-3′) and B1392R (5′-CAG GGG CGG TGT GTA-3′) using the following conditions: one initial denaturation at 95°C for 3 min, 26 cycles at 94°C for 30 s, 57°C for 45 s, and 72°C for 1 min, and a final extension at 72°C for 10 min. Methanol dehydrogenase genes were amplified with the primers *mxaFxoxF*f916 and *mxaFxoxF*r1360 (Table 1) designed to preferentially amplify *mxaF*, *xoxF4*, and *xoxF5*, using the following conditions: one initial denaturation at 95°C for 3 min, 35 cycles at 94°C for 20 s, 55°C for 45 s, and 72°C for 1 min, and one final extension at 72°C for 10 min. The design of the primers *mxaFxoxF*f916 and *mxaFxoxF*r1360 was based on the alignments of the *mxaF*, *xoxF4*, and *xoxF5* public sequences. The alignments of other *xoxF* subfamilies did not show sufficiently long conserved regions for designing potentially acceptable primers. Sanger DNA sequencing were performed at the Instituto de Biotecnología (UNAM, www.ibt.unam.mx) with the primers used for PCR amplification.

### Sequence analysis

Sequence analyses were performed with MEGA 7.0 (32). The sequences were aligned with the database sequences of related microorganisms by ClustalW. Pairwise distances and neighbor joining trees were used to elucidate the genus identity of the 16S rRNA sequences. The phylogeny of methanol dehydrogenases was inferred with the maximum likelihood method with the deduced amino acid sequences. Initial trees were assessed by applying Neighbor-Join and BioNJ algorithms to a matrix of pairwise distances, and then selecting the topology with the greatest log likelihood value. Confidence was evaluated by bootstrapping with 500 iterations.

### Nucleotide sequences

16S rRNA sequences have been deposited in GenBank under the accession numbers KT936080–KT936091, KT936093, KT936095, KT936096, KT936105, KT936109–KT936114, KT936119, KT936125–KT936127, KT936134, KT936135, KT936140, KT936141, KT936144, KT936145, and KY00648–KY00653; and *xoxF* sequences under the accession numbers KT932117–KT932121, KT932123, KT932124, KT932126–KT932128 and KY884986–KY884988 (Table 2).

## Results

Thirty-eight bacterial isolates (Classes *Alphaproteobacteria*, *Betaproteobacteria*, *Gammaproteobacteria*, *Actinobacteria*, and *Sphingobacteriia*) were obtained using methanol as the sole carbon and energy sources (Table 2). All isolates showed facultative growth using other carbon and energy sources. No obligate methylotrophic bacteria were found. Twenty-two strains were isolated from the plant surface (one *Actinobacteria*, four *Alphaproteobacteria*, four *Betaproteobacteria*, and thirteen *Gammaproteobacteria*); six isolates were endophytic (three *Alphaproteobacteria* and three *Gammaproteobacteria*); and ten were rhizospheric (two *Actinobacteria*, one *Sphingobacteriia* (Phylum *Bacteroidetes*), four *Alphaproteobacteria*, and three *Gammaproteobacteria*). The identity of methylotrophic bacteria from the plant surface, from inside the plant, or the rhizosphere were as follows: *Arthrobacter*, one epiphyte and two rhizospheric; *Pedobacter*, one rhizospheric, *Microvirga*, four epiphytes; *Inquilinus*, two rhizospheric; *Methylobacterium*, one epiphyte, and one rhizospheric; *Rhizobium*, one rhizospheric; *Sphingomonas*, one endophyte; *Subaequorebacter*/*Geminicoccus*, one endophyte; *Massilia*, four epiphytes; *Acinetobacter*, twelve epiphytes, three rhizospheric, and three endophytes; and *Pseudomonas*, one epiphyte (Table 2, Fig. S1).

All methylotrophic isolates tested showed growth with methanol as the carbon and energy sources and Ca^2+^ or REE, Ce^3+^, as co-factors (Table 3). Different isolates showed distinct methylotrophic growth rates. Hence, the time of their maximum growth in the presence of Ce^3+^ ranged between a 72- and 144-h incubation. Most of the isolates did not show any preference for either co-factor, whereas it was apparent for some that one of the co-factors improved methylotrophic growth. In this assay, 22 isolates were selected to include all taxonomical groups. These strains included two *Actinobacteria*, one *Sphingobacteriia*, ten *Alphaproteobacteria*, one *Betaproteobacteria*, and eight *Gammaproteobacteria*.

**Fig. 1:**
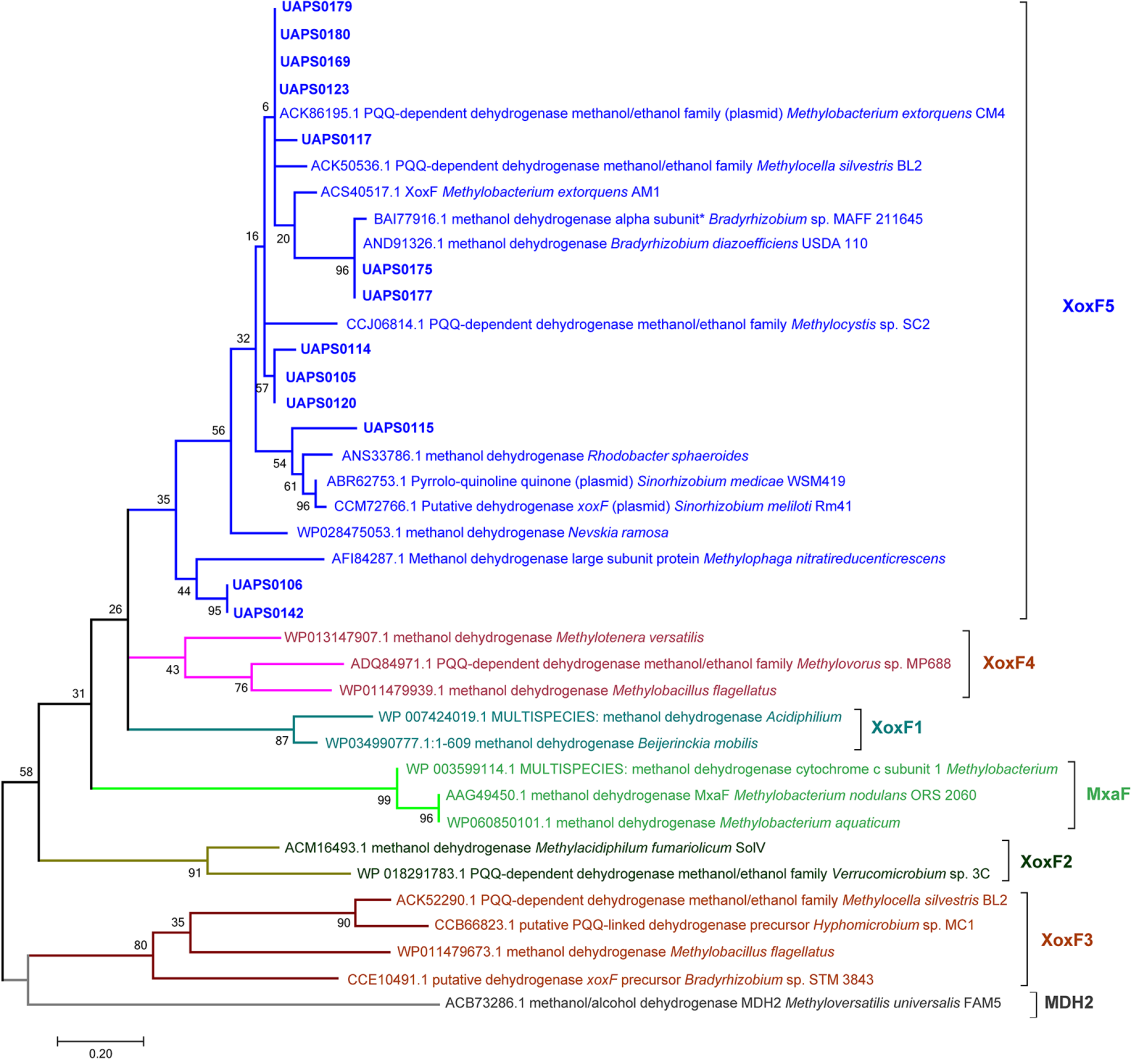
Phylogeny of putative methanol dehydrogenase amplicons of *N. macrocephala* isolates. Sequences of *N. macrocephala* isolates are shown in bold blue letters. Sequences were aligned by Muscle. Phylogeny was constructed with maximum-likelihood in MEGA 6.0 using deduced amino acid sequences. A total of 500 iterations were used for bootstrapping.

**Fig. 2:**
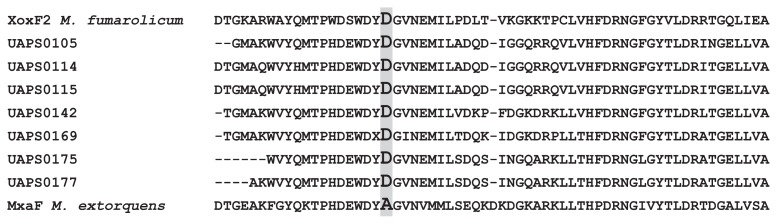
Partial alignment of sequences of methanol dehydrogenases that cover the region encoding Asp^301^. Asp^301^ (D) has been detected in all XoxF dehydrogenases and it is necessary for REE coordination. MxaF dehydrogenases do not possess Asp^301^.

Amplicons (approximately 550 bp in length) with *mxaFxoxF*targeted primers were obtained in 34.2% (13) of the isolates. All sequences were more similar to XoxF-like methanol dehydrogenases than to MDH-like methanol dehydrogenases (Fig. 1). After the sequence analysis, five *Alphaproteobacteria*, three *Betaproteobacteria*, four *Gammaproteobacteria*, and one *Actinobacteria* isolates were found to possess *xoxF5*-like sequences. Furthermore, Asp^301^ characteristic of XoxF dehydrogenases was detected in all of the amplicons that covered that region (Fig. 2, Table 2).

**Fig. 3:**
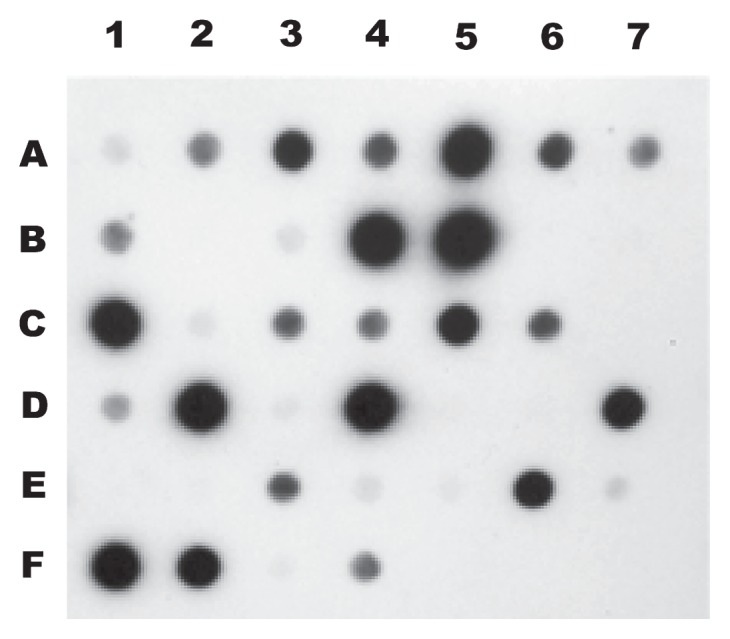
Dot-blot hybridization with *xoxF*. Lines A1, UAPS0104; A2, UAPS0105; A3, UAPS0106; A4, UAPS0181; A5, UAPS0110; A6, UAPS0102; A7, UAPS0184; B1, UAPS0182; B2, UAPS0149; B3, UAPS0121; B4, UAPS0122; B5, UAPS0123; B6, UAPS0126; B7, UAPS0127; C1, UAPS0114; C2, UAPS0136; C3, UAPS0137; C4, UAPS0180; C5, UAPS0115; C6, UAPS0142; C7, UAPS0145; D1, UAPS0179; D2, UAPS0174; D3, UAPS0118; D4, UAPS0155; D5, UAPS0156; D6, UAPS0158; D7, UAPS0177; E1, UAPS0165; E2, UAPS0120; E3, UAPS0160; E4, UAPS0168; E5, UAPS0169; E6, UAPS0175; E7, UAPS0172; F1, UAPS0117; F2, UAPS0183; F3, UAPS0163; F4, *M. extorquens* JCM2802 (100 ng); F5, *Ustilago maydis* 207; F6 and F7, void. One microgram of total DNA of the bacterial strains evaluated was transferred to nylon membranes. PCR probes (100 ng) were obtained by the PCR amplification of *Methylobacterium extorquens* JCM2802 with the primers xoxF5f361 5′-CAG GAT CCG TCC GTG AT-3′ and xoxF5r603 5′-SGA GAT GCC GAC GAT GA-3′.

Among the twenty-five isolates from which amplicons were not obtainable with the *mxaf* and *xoxF*-targeted primers, eleven clearly hybridized with a *xoxF5* probe from *M. extorquens* (Table 2; Fig. 3): one *Actinobacteria*, four *Alphaproteobacteria*, one *Betaproteobacteria*, and five *Gammaproteobacteria*. The remaining fourteen isolates did not hybridize to the *xoxF5* probe or were not amplified with the *mxaFxoxF* primers, including one *Actinobacteria*, one *Sphingobacteriia*, two *Alphaproteobacteria*, and ten *Gammaproteobacteria*. Hybridization with the *mxaF* probe was very faint; however, some dots indicated that the organism possessed *mxaF* loci (Fig. S2).

## Discussion

Methanol and methane are very common carbon compounds produced by plants (19, 28). Methylotrophy is distributed in many different taxa (31). In this study, bacteria of the Classes *Actinobacteria*, *Sphingobacteria*, *Alpha*-, *Beta*-, and *Gammaproteobacteria* were isolated in a methanol-based medium. Since this mostly plant-originated compound is a very common C-source in nature, numerous plant-associated microorganisms have the capability to use it.

Among the methylotrophs cultivated from *N. macrocephala* and its rhizosphere, most were isolated from the stem surface. We hypothesize that this relates to the presence of stomata and consequently to the main source of methanol from inner plant tissues (19). All the dehydrogenase sequences obtained were similar to *xoxF5*, genes that are phylogenetically related to other *xoxF* subfamilies and to *mxaF*. These *xoxF5*-like sequences were obtained from isolates belonging to the Classes *Actinobacteria*, and *Alpha*-, *Beta*-, and *Gammaproteobacteria*. *mxaF*-like sequences were previously identified in these classes and the phyla *Bacteroidetes* and *Verrucomicrobia* (4, 9, 29). Aspartic acid 301, the amino acid responsible for REE coordination (27), was detected in all of the sequences that covered that region. In contrast, none of the sequences showed different amino acids to Asp in that position. Additionally, none of the amplicons with *mxaFxoxF*targeted primers were *mxaF*; they were *xoxF5*. Therefore, the sequenced amplicons coded for XoxF dehydrogenases. Nevertheless, we cannot rule out that some of the isolates possessed *mxaF* due to faint dot-blot hybridization with a *mxaF* probe. Positive hybridization with the *xoxF* probe indicated that these strains may possess *xoxF5*. Although we cannot exclude sequences of other *xoxF* subfamilies cross-hybridizing with the probe, hybridization and washing stringency conditions reduce that possibility. Some of the isolates that did not hybridize with the *xoxF5* and *mxaF* probes or were not amplified with *mxaF-xoxF* primers may possess other sequences of the *xoxF* subfamilies or other methanol dehydrogenases such as MDH2 or NAD-dependent methanol dehydrogenase. Although we also designed primers and unsuccessfully attempted the amplification of methanol:NDMA oxidoreductase (Table S1, Results not shown), its presence cannot be excluded. In some of the cases in which we detected hybridization to *mxaF* or *xoxF5*, we did not obtain amplicons of methanol dehydrogenase genes. This inconsistency may be related to the design of the primers. All isolates tested in the methylotrophy assay grew using Ce^3+^, as expected, but also used Ca^2+^ as a co-factor. Therefore, it currently remains unclear whether XoxF enzymes accept Ca^2+^ besides REE, as suggested by Keltjens *et al.* 2014 (27).

The ubiquities of *xoxF*, of their peptides, and of the bacteria carrying them in nature have been demonstrated in different studies, including the *N. macrocephala*-related ecosystem. XoxF has been detected in the phyllospheres of rice, clover, soybean, and *Arabidopsis* (15, 30). A previous study in a particular marine environment also showed the high abun-dance of XoxF (53). In an autecological approach, a semi *in situ* SIP assay detected the strong expression of a *xoxF*-like locus in *Methylotenera mobilis* (59). Furthermore, methanol oxidation in *Methylomicrobium buryatense*, possessing *xoxF* and *mxaFI* functional loci appeared to be mainly accomplished by XoxF (12).

It has not yet been established whether there is a biogeography of subfamilies of *xoxF*. New studies on methylotrophy with non-culture and culture approaches in different environments are needed. A pioneer ecological study of the different *xoxF* subfamilies in coastal marine water only detected sequences of the clusters *xoxF4* and *xoxF5* (55). In a different environment, the methanol dehydrogenase peptides XoxF and MxaF of *Methylobacterium*, a microorganism that only possesses *xoxF5* and *mxaF* sequences, were abundantly detected in the phyllosphere of soybean, clover, rice, and *A. thaliana* (15, 30). The present culture-dependent study demonstrated the presence of microorganisms possessing sequences of the subfamily *xoxF5* in the semi-arid environment of *N. macrocephala*.

A previous study with some XoxF enzymes reported high affinity for methanol (27, 50). If the enzymes of more diverse microorganisms exhibit similar behaviors, XoxF may be crucial for methylotrophic bacteria that thrive in plants showing slow metabolic properties and producing methanol at low rates, such as cacti. The presence of XoxF may be favored in environments in which sand, and, thus, REEs, are abundant, such as arid lands (50).

Besides its participation in methylotrophic metabolism, XoxF may be involved in the regulation of stress responses and in denitrification metabolism (17, 45). Its putative role in stress responses may be particularly important in semi-arid areas and in plant surfaces.

Although the typical methanol dehydrogenase from *Actinobacteria* is methanol:NDMA oxidoreductase, they do not exclusively carry it. The synthesis of PQQ by *Actinobacteria* in the presence of methanol suggested the presence of a PQQ-dependent methanol dehydrogenase (22). In another study, a *Brevibacterium casei* strain, an actinobacterial methylotrophic human mouth microorganism, carried a *mxaF* methanol dehydrogenase sequence (4; see Fig. 1), and more recently, metagenomic studies in the desert of Atacama detected *Pseudonocardia* PQQ methanol dehydrogenase genes (36). The presence of *xoxF* genes in *Actinobacteria* isolated in this study may have originated from lateral transfer events, as has been detected in the locus *mxaF* of methanotrophic bacteria (7, 33) and in methylotrophic *Alphaproteobacteria* (7).

The methylotrophic isolates from the environment of *N. macrocephala* belonged to *Proteobacteria*, *Actinobacteria*, and *Sphingobacteriia*. Among them, *Acinetobacter* spp. (*Gammaproteobacteria*) were the most frequently isolated organisms. It has been reported that *Acinetobacter* uses methanol as a carbon source (20, 61) and a methanol dehydrogenase sequence coding Asp^301^ has previously been detected in this genus (20). Similar to these findings, other studies identified *Proteobacteria* and *Actinobacteria* as some of the most common taxa in the rhizosphere and soil from cacti and other plants from arid lands (2, 11, 13, 26).

Methylotrophic bacteria are ubiquitous and have meaningful roles in ecosystems. Since water is mostly limited in arid environments, perennial plants from these environments show restrained growth, particularly throughout the dry season. The community of methylotrophic culturable bacteria associated with the semi-arid thriving cactus *N. macrocephala* include *xoxF*-like dehydrogenases-possessing microorganisms. Their ecological role in xerophytic plants warrants further study. Since the cultivation procedures employed in the present study do not necessarily produce a real picture of bacterial diversity, the future application of non-culture approaches will enrich knowledge on methylotrophic diversity in this environment. In future inoculation experiments, we intend to detect the isolates of methylotrophic bacteria that may stimulate the growth of *N. macrocephala*, particularly in the vulnerable juvenile stage.

## Supplementary Material



## Figures and Tables

**Table 1:** Methanol dehydrogenase primers.

Primer*	Sequence (5′-3′)	Target	Reference
mxa f1003	GCG GCA CCA ACT GGG GCT GGT	*mxaF*	(42)
mxa r1561	GGG CAG CAT GAA GGG CTC CC		
*xoxF*361f	CAG GAT CCG TCC GTG AT	*M. extorquens xoxF*	This work
*xoxF*603r	SGA GAT GCC GAC GAT GA		
*mxaFxoxF*916f	GGC GAC AAC AAG TGG WCG ATG	*mxaF*, *xoxF4*, *xoxF5*	This work
*mxaFxoxF*1360r	AGT CCA TGC AGA CRT GGT T		

* Numbers indicate approximate position in the gene.

**Table 2:** Methylotrophic culturable isolates from *N. macrocephala*.

Isolate	Genus	Taxonomic Class	16S rRNA Acc. Num.	Origin	Hybridization with		Amplicons with *mxaF-xoxF* primers Acc. Num.	Subjected to the methanol-Ca^2+^/Ce^3+^ experiment

					*mxaF*	*xoxF*		
UAPS0102	*Arthrobacter*	Actinobacteria	KT936093	Rhizospheric	ND	P	NA	Yes
UAPS0104	*Arthrobacter*		KT936095	Epiphytic	ND	S	NA	No
UAPS0105	*Arthrobacter*		KT936096	Rhizospheric	ND	P	KT932119^D^	Yes
UAPS0126	*Pedobacter*	*Sphingobacteriia*	KT936125	Rhizospheric	ND	N	NA	Yes
UAPS0120	*Microvirga*	Alphaproteobacteria	KT936105	Epiphytic	ND	N	KY884987	Yes
UAPS0121	*Microvirga*		KT936119	Epiphytic	S	S	NA	Yes
UAPS0136	*Microvirga*		KT936112	Epiphytic	ND	S	NA	Yes
UAPS0137	*Microvirga*		KT936113	Epiphytic	ND	P	NA	Yes
UAPS0106	*Inquilinus*		KT936134	Rhizospheric	N	P	KY884986	Yes
UAPS0142	*Inquilinus*		KT936135	Rhizospheric	P	P	KT932126^D^	Yes
UAPS0122	*Methylobacterium*		KT936114	Endophytic	P	P	NA	Yes
UAPS0123	*Methylobacterium*		KT936111	Rhizospheric	S	P	KY884988	Yes
UAPS0160	*Rhizobium*		KT936127	Rhizospheric	P	P	NA	No
UAPS0110	*Sphingomonas*		KT936140	Endophytic	N	P	NA	No
UAPS0115	*Subaequorebacter*/	KT936141	Endophytic	N	P	KT932127^D^	Yes	
	*Geminicoccus*							
UAPS0114	*Massilia*	Betaproteobacteria	KT936109	Epiphytic	P	P	KT932123^D^	No
UAPS0174	*Massilia*		KT936144	Epiphytic	P	P	NA	No
UAPS0175	*Massilia*		KT936145	Epiphytic	S	P	KT932128^D^	No
UAPS0177	*Massilia*		KT936110	Epiphytic	N	P	KT932124^D^	Yes
UAPS0117	*Acinetobacter*	Gammaproteobacteria	KT936080	Epiphytic	S	P	KT932117	Yes
UAPS0118	*Acinetobacter*		KT936081	Epiphytic	P	S	NA	No
UAPS0127	*Acinetobacter*		KT936082	Epiphytic	ND	N	NA	No
UAPS0145	*Acinetobacter*		KT936083	Epiphytic	P	N	NA	No
UAPS0149	*Acinetobacter*		KT936084	Epiphytic	P	N	NA	No
UAPS0156	*Acinetobacter*		KT936085	Epiphytic	P	N	NA	No
UAPS0158	*Acinetobacter*		KT936086	Rhizospheric	P	N	NA	No
UAPS0163	*Acinetobacter*		KT936087	Epiphytic	P	N	NA	Yes
UAPS0165	*Acinetobacter*		KT936088	Epiphytic	ND	N	NA	No
UPAS0168	*Acinetobacter*		KT936089	Epiphytic	P	S	NA	No
UAPS0169	*Acinetobacter*		KT936090	Epiphytic	S	S	KT932118^D^	No
UAPS0172	*Acinetobacter*		KT936091	Epiphytic	P	S	NA	No
UAPS0179	*Acinetobacter*		KY400648	Rhizospheric	ND	S	KT932120	Yes
UAPS0180	*Acinetobacter*		KY400649	Endophytic	ND	P	KT932121	Yes
UAPS0181	*Acinetobacter*		KY400650	Epiphytic	ND	P	NA	No
UAPS0182	*Acinetobacter*		KY400651	Endophytic	ND	P	NA	Yes
UAPS0183	*Acinetobacter*		KY400652	Endophytic	ND	P	NA	No
UAPS0184	*Acinetobacter*		KY400653	Rhizospheric	ND	P	NA	No
UAPS0155	*Pseudomonas*		KT936126	Epiphytic	P	P	NA	Yes

N, negative hybridization; P, positive hybridization; S, slight hybridization; ND, not determined; NA, not amplificated with the primers*mxa*f916 and *mxa*r1360 D, XoxF sequences long enough to cover Asp301.

**Table 3:** Methylotrophic growth with Ca^2+^ or Ce^3+^ as co-factor for methanol dehydrogenase.

Time	Genus	Strain	Growth with	

			Ca^2+^	Ce^3+^
72 h	*Sphingomonas*	UAPS0110	0.7883	0.7637
	*Methylobacterium*	UAPS0123	1.0710^*^	0.7660
	*Rhizobium*	UAPS0160	0.8717	0.9367

96 h	*Methylobacterium*	UAPS0122	0.4123	0.3007

120 h	*Arthrobacter*	UAPS0102	0.8563	1.3483^*^
	*Arthrobacter*	UAPS0105	0.7910	1.1037
	*Subaequorebacter*/			
	*Geminicoccus*	UAPS0115	0.2057	0.7513^*^
	*Acinetobacter*	UAPS0117	1.0703^*^	0.7873
	*Microvirga*	UAPS0120	0.9390^*^	0.4777
	*Microvirga*	UAPS0121	0.9967^*^	0.7640
	*Pedobacter*	UAPS0126	0.9957^*^	0.7133
	*Microvirga*	UAPS0137	1.1033	0.8533
	*Inquilinus*	UAPS0142	0.6683	0.9637
	*Pseudomonas*	UAPS0155	0.6140	0.6230
	*Acinetobacter*	UAPS0163	1.2073	1.2163
	*Acinetobacter*	UAPS0169	0.9680	1.4060^*^
	*Massilia*	UAPS0177	0.6817	1.0697^*^
	*Acinetobacter*	UAPS0180	1.3707	1.1673
	*Acinetobacter*	UAPS0182	0.9920	1.0623
	*Acinetobacter*	UAPS0183	0.8610	1.1247

144 h	*Microvirga*	UAPS0136	0.6087	0.2263
	*Acinetobacter*	UAPS0179	0.3757	0.8020

Data correspond to absorbance at 600 nm, the media of three replicates. Cells were incubated under shaking at 30°C. The registers correspond to their time of maximum growth in the presence of Ce^3+^. The growth of each strain in the presence of Ca^2+^/Ce^3+^ wascomparedandthesignificance of differences between two values was assessed by the unpaired t-test, *P*>0.05. Values marked with an asterisk are significantly higher than their counterparts.
