# Supplementary material for: Culturable Facultative Methylotrophic Bacteria from the Cactus Neobuxbaumia macrocephala Possess the Locus xoxF and Consume Methanol in the Presence of Ce3+ and Ca2+
Source: Microbes Environ. 2017 Sep 27;32(3):244–51. doi: 10.1264/jsme2.ME17070 (PMC5606694; doi:10.1264/jsme2.ME17070)
Supplement: Supplementary file 1 [file 32_244_s1.pdf]

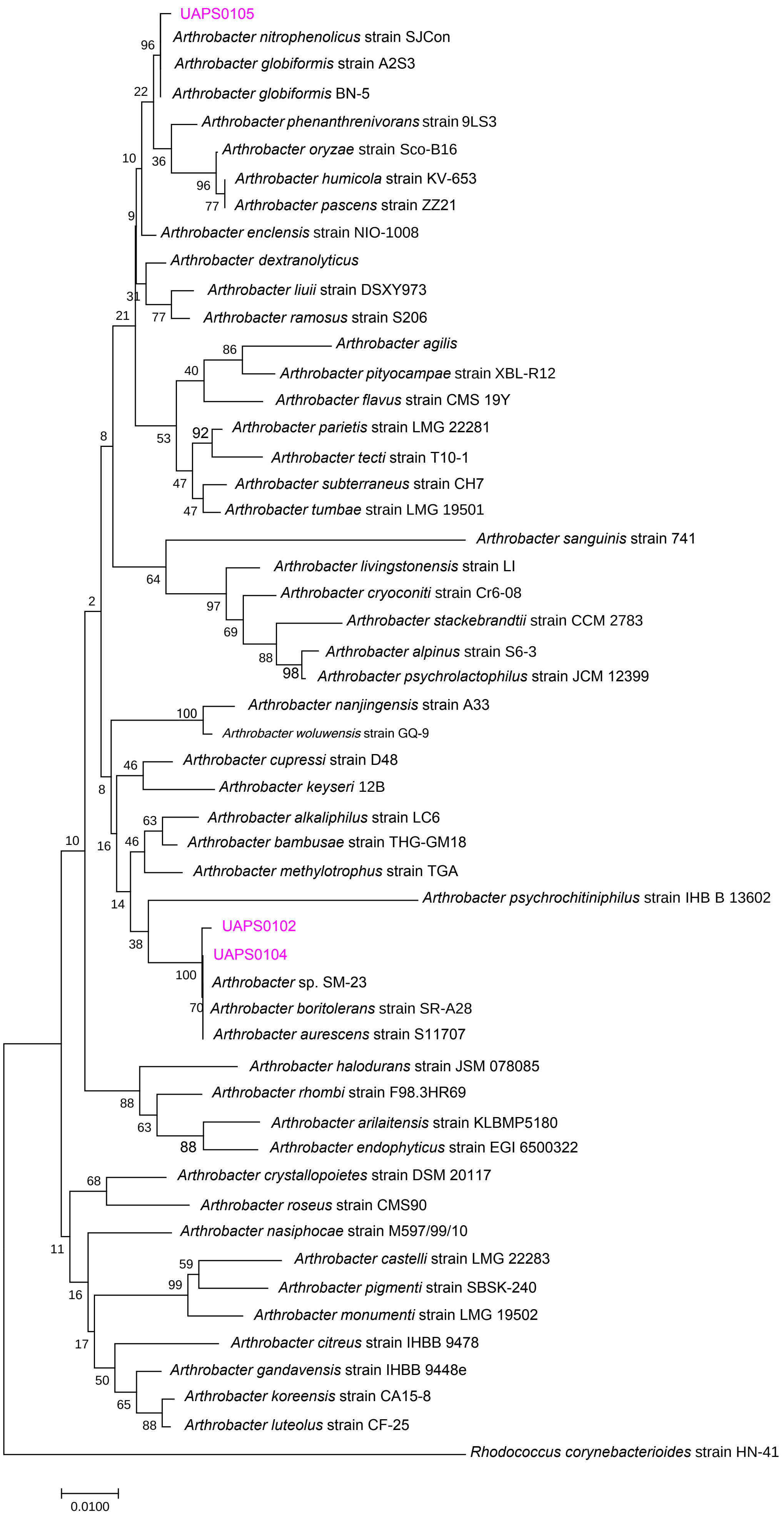

Fig. S1 16S taxonomical identity of Class *Actinobacteria* isolates

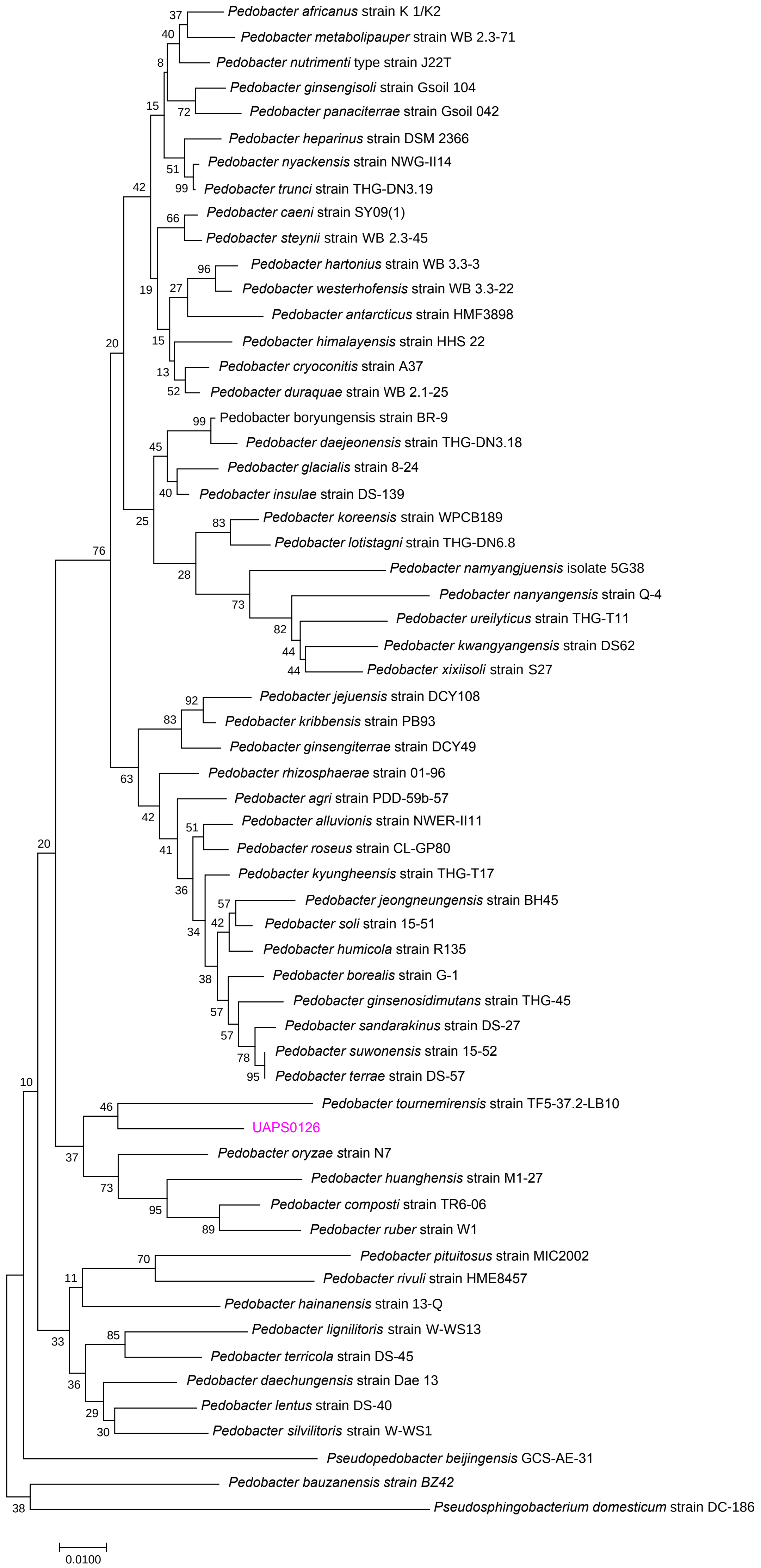

Fig. S1. 16S taxonomical identity of the Class *Sphingobacteriia* isolate

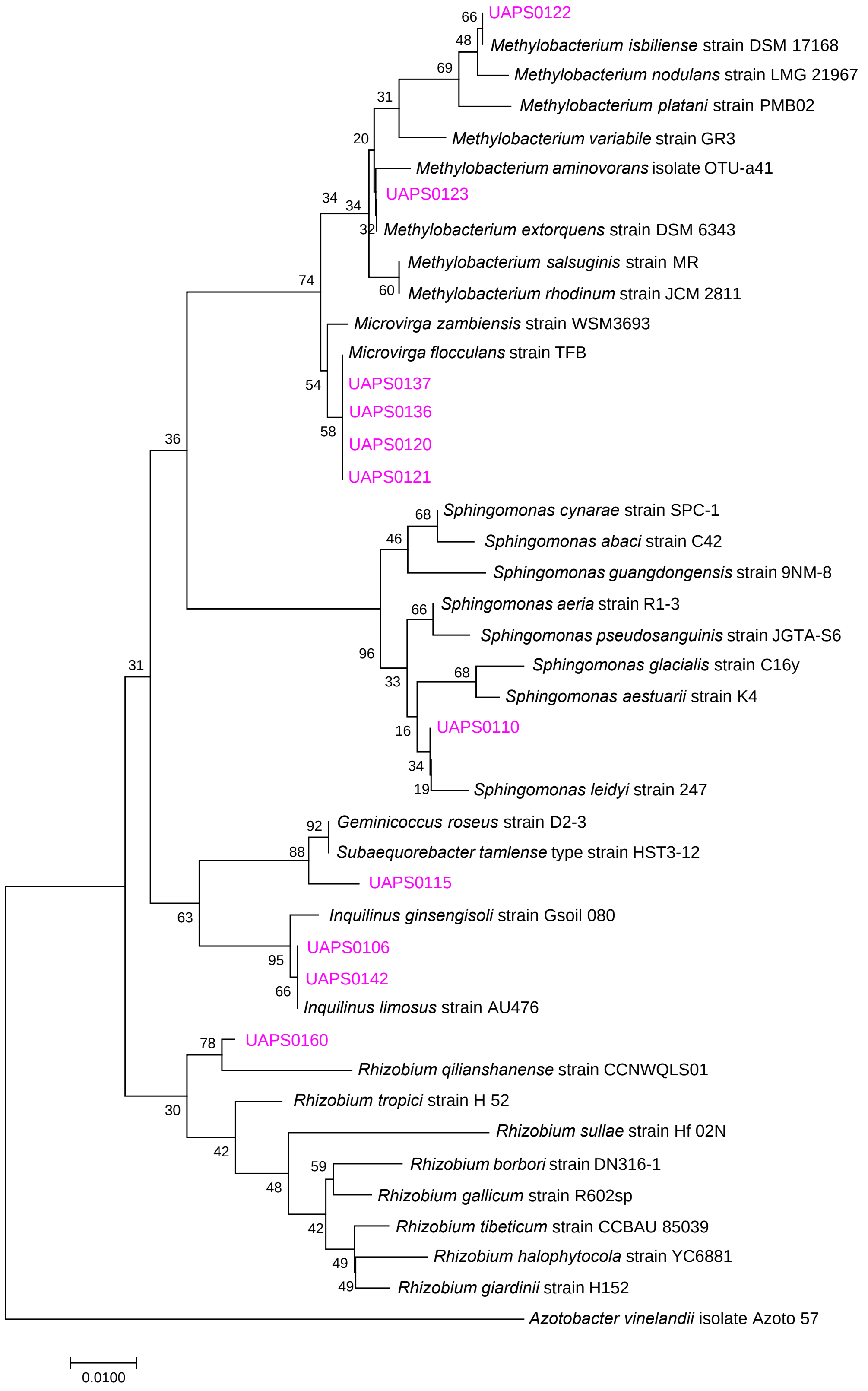

Fig. S1. 16S taxonomical identity of Class *Alphaproteobacteria* isolates

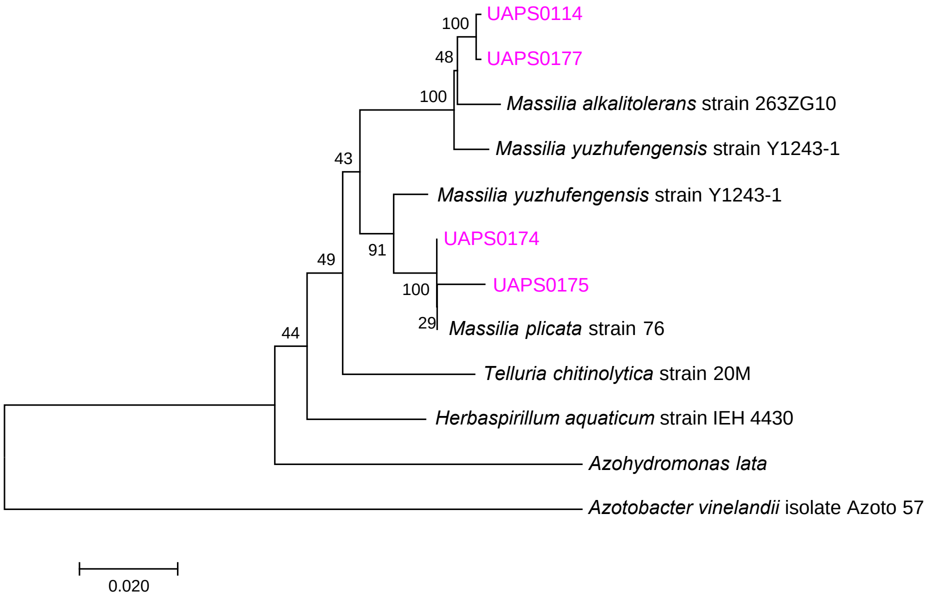

Fig. S1. 16S taxonomical identity of Class *Betaproteobacteria* isolates

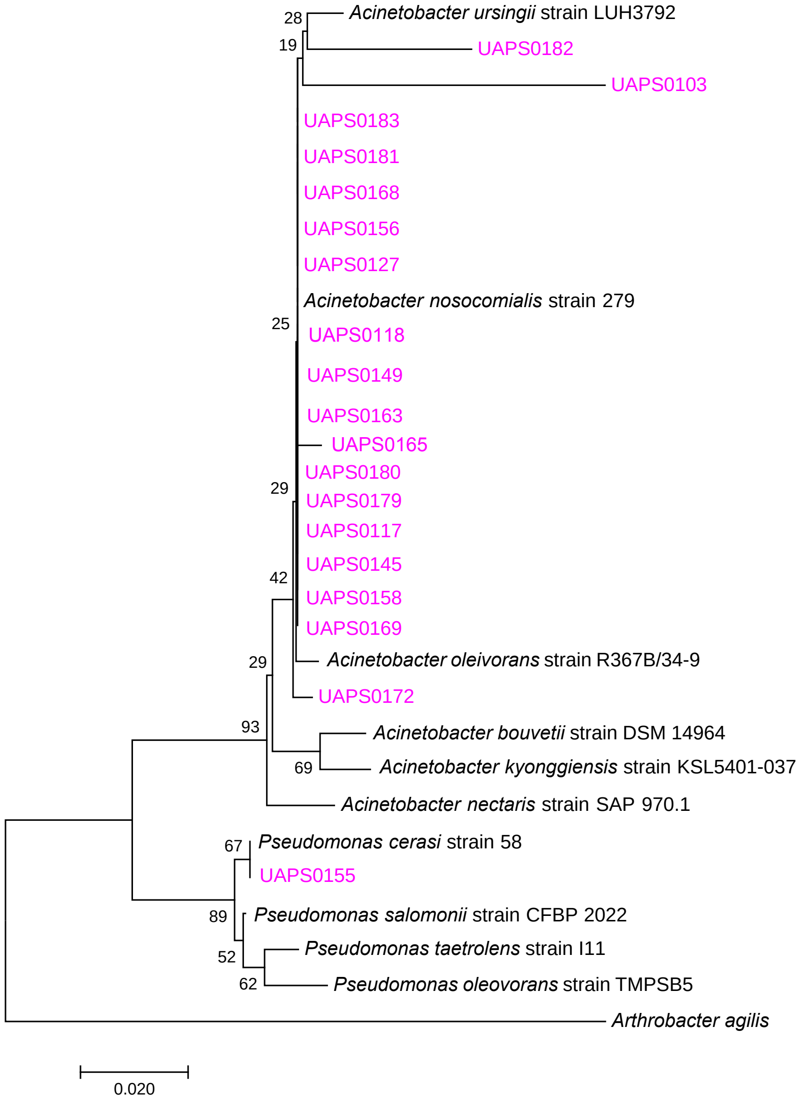

Fig. S1. 16S taxonomical identity of Class *Gammaproteobacteria* isolates

mxaF DOT BLOT ARRANGMENT

C+	blank	77	75	74
72	Ni	69	68	Ni
63	60	58	56	55
49	45	42	Ni	23
22	21	18	17	15
14	Ni	10	06	C-

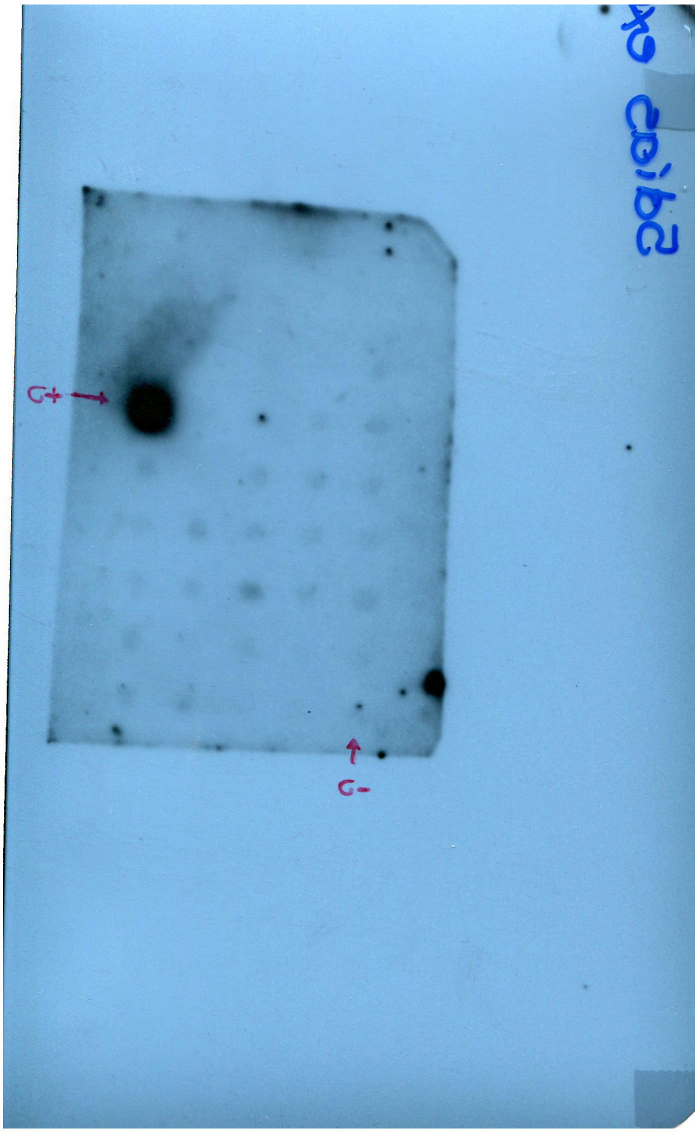

Positive control **C+** *Methylobacterium extorquens* JCM2802.

Negative control **C-** *U. maydis 207*.

Numbers indicate the reduced name of the isolates. For example, 77 means UAPS07077.

Ni, strain not included in this work.

Fig. S2. *mxaF* dot blot hybridization

Table S1. Methanol:NDMA (N,N'-dimethyl-4-nitrosoaniline oxidorreductase primers.

Primer	Sequence (5'-3')
NDMAY270F	YCC CAA GGA CTA CAA CGT CAT
NDMAGA270F	CCA AGG ACT ACA ACG TCA TGG A
NDMA270F	CCC AAG GAC TAC AAC GTC AT
NDMA850R	CCS AGN CCR CCS GAG TTG AA
